# Revisiting Grammatical Complexity in L2 Writing *via* Exploratory Factor Analysis

**DOI:** 10.3389/fpsyg.2022.860753

**Published:** 2022-06-30

**Authors:** Ge Lan, Xiaorui Li, Qiusi Zhang

**Affiliations:** ^1^Department of English, City University of Hong Kong, Hong Kong, Hong Kong SAR, China; ^2^Oral English Proficiency Program, Purdue University, West Lafayette, IN, United States

**Keywords:** grammatical complexity, L2 writing, writing assessment, writing development, grammatical measures

## Abstract

Since the 1990s, grammatical complexity has received substantial research attention in applied linguistics (Bulté and Housen, 2014). The representation of grammatical complexity has expanded in L2 writing with the application of diverse measures in empirical studies in the recent three decades (1991–2020). In response to this situation, we found it important to revisit grammatical complexity, and an exploratory factor analysis was applied to explore latent dimensions (i.e., factors) of grammatical complexity in L2 writing. We analyzed Lu’s (2011) 14 grammatical complexity measures in the L2 corpus of the British Academic Written English Corpus. We then proposed a four-factor model with “clausal subordination,” “phrasal construction”, “global length unit” and “others.” The four factors generally align with the types of grammatical complexity proposed in Lu (2011), but differences on six measures are also found. Noteworthy points were discussed to interpret the reasons behind the differences. Research implications are provided to show further research directions.

## Introduction

Grammatical complexity has been considered as an important construct in applied linguistics, and it has received growing research attention in varied areas, for instance, second language acquisition (SLA) and second language (L2) writing (Bulté and Housen, 2012). A wide range of grammatical complexity measures have been applied in relevant empirical studies to explore different components of grammatical complexity (e.g., clausal complexity and noun phrase complexity). This leads to an expansive representation of this construct in applied linguistics. While acknowledging the importance of such expansion, we consider it important to revisit the representation of grammatical complexity from an inductive perspective. We then asked ourselves the following question: Do existing popular measures of grammatical complexity appropriately reflect the latent dimensions of this construct? To answer this question, the purpose of this study is to explore the possible latent dimensions of grammatical complexity with an exploratory factor analysis (EFA).

### Grammatical Complexity in L2 Writing Research

Grammatical complexity has been found to be important due to its relationship to multiple core constructs in L2 writing research, for instance writing development, writing proficiency, and writing quality. Wolfe-Quintero et al. (1998) provided the initial in-depth review of grammatical complexity measures, and from then on, scholars have increasingly applied the measures to investigate many variables related to L2 writing, such as written topics, writing scores, written genres (e.g., Yoon, 2017; Kyle and Crossley, 2018). The measures of grammatical complexity can be primarily summarized into three categories: (a) specific lexico-grammatical features (e.g., relative clauses, prepositional phrases), (b) large-grained syntactic measures (e.g., mean length of T-units), and (c) fine-grained syntactic measures (e.g., adjectival modifiers per nominal subject). For the sake of convenience in discussion, we use the term, grammatical complexity, to refer to relevant research based on the application of all the three types of measures.

In the recent decades, grammatical complexity studies have been influenced by specific seminal publications. For example, Biber et al. (2011) proposed a hypothesized index of writing complexity features, and the index includes three types of grammatical forms, namely finite dependent clauses, non-finite dependent clauses, and dependent phrases. The grammatical forms also include their corresponding grammatical functions, which are adverbials, complements, and noun modifiers. Altogether the index contains 28 grammatical features. There are five developmental stages in the index, and each is associated with certain sets of grammatical features. Many recent studies have been conducted based on the index to investigate grammatical complexity in L2 writing (e.g., Parkinson and Musgrave, 2014; Staples et al., 2016; Lan et al., 2022).

Also, Lu (2011) summarized a model based on 14 measures of grammatical complexity, which were frequently applied in previous studies. Lu’s (2011) model includes specific grammatical types of the 14 measures: length of production (e.g., mean length of T-unit), subordination (e.g., dependent clauses per T-unit), coordination (e.g., coordinate phrases per clause), sentence complexity (i.e., clauses per sentence), and particular structures (e.g., complex nominals per T-unit). The calculation of these 14 grammatical complexity measures can be automated with the application of the L2SCA (Lu, 2014). Recent studies based on these 14 measures also focus on multiple factors that influence writing development, for instance, written gernes (e.g., Yoon and Polio, 2017), academic levels (e.g., Lu, 2011), and writing quality (e.g., Casal and Lee, 2019).

### Grammatical Complexity and Exploratory Factor Analysis

Exploratory factor analysis is a statistical technique that aims to demonstrate relationships among variables and possible latent dimensions (Tabachnick and Fidell, 2013). EFA has not been substantially applied to analyze grammatical complexity, and a possible reason behind this is that EFA is an advanced statistical technique. In applied linguistics, we only found a few studies that applied EFAs to systematically explore latent dimensions of L2 complexity. Yoon (2017) analyzed linguistic complexity in L2 writing, and a part of Yoon’s aims is to explore whether the measures of lexical complexity, grammatical complexity and morphological complexity tap into different dimensions of complexity. With the application of an EFA, Yoon (2017) showed that (a) “lexical and morphological dimensions of complexity loaded on one construct” and (b) “the unit-length measures with different base units loaded on different constructs” (p.130). Also, Li and Zhang (2020) studied the latent structure of L2 linguistic complexity by using an EFA followed by a CFA in writing tasks from Chinese EFL students. A set of measures were included, including subordination measures, phrasal measures, lexical measures, and overall complexity measures. They confirmed that the multidimensionality of L2 linguistic complexity and presented three latent components, which were clausal complexity, phrasal complexity, and lexical complexity. Next, Jiang et al. (2021) investigated the relation between linguistic complexity (i.e., lexical complexity, syntactic complexity and phraseological complexity) and human rater’s overall judgment on writing quality with a learner corpus of research papers. Their EFA findings indicated that phraseological complexity measures were tapped into an individual latent construct, which should be considered an independent construct of L2 complexity, especially for rating writing quality of student papers.

### Research Gaps and Research Questions

Having said this, it is important to follow the recent research trend to explore latent dimensions of grammatical complexity. In response to Polio’s (2017) calling for further investigation on validating the existing measures of complexity, accuracy and fluency in L2 writing research, this study will add more empirical evidence of how grammatical complexity has been represented in L2 writing. This is essential because the representation of this construct has been expanded over the past three decades (1990–2020). Thus, we revisited the 14 frequently used measures of grammatical complexity in Lu (2011). This EFA model is an objective presentation on if they accurately measure the grammatical structures that we expect them to measure. This EFA model will provide insights on the application of grammatical measures in empirical studies of L2 writing development in the future. In particular, our study aims to answer two research questions:

1. What are the latent dimensions of grammatical complexity?
2. Are the dimensions in our EFA model consistent with the proposed grammatical types in Lu’s (2011) model?

## Methods

### Corpus

The corpus of this study is a subset of the British Academic Written English Corpus (BAWE). This BAWE corpus contains diverse written genres (e.g., essays, research reports) across four disciplinary domains (e.g., Arts and Humanities, Life Sciences). We extracted all L2 academic papers from BAWE to represent L2 writing in this study. Our corpus contains 823 files with 2,043,484 tokens and a mean text length of 2,483 tokens. The files were produced by L2 students at four academic levels: first/second/third-year undergraduate (i.e., level-1, level-2, and level-3) and the graduate level (i.e., level-4). We consider the corpus to comprehensively represent L2 academic writing.

Table 1 shows the basic information of the L2 corpus. Level-1 includes 167 files; level-2 includes 150 files; level-3 includes 181 files; level-4 includes 325 files (see Table 1 for more details). Next, the L2SCA was used to automate the calculation on 14 measures of grammatical complexity based on the L2 corpus. Then, a dataset was built based on the values of the 14 measures from the L2SCA in Statistics Package for Social Sciences (SPSS). By the end of this step, we consider that the dataset is ready to run the EFA.

**TABLE 1:** Description of the L2 writing corpus.

Level	Number of texts	Tokens	Mean text length
1	167	281,302	1,684
2	150	324,413	2,163
3	181	449,366	2,483
4	325	988,403	3,041
Total	823	2,043,484	2,483

### Procedures of Exploratory Factor Analysis

Exploratory factor analysis was performed on the dataset to explore the latent dimensions of grammatical complexity. Step-1 is checking factorability and sampling adequacy. We examined descriptive statistics and conducted the Kaiser-Meyer-Olkin (KMO) test and Bartlett’s Test of Sphericity to confirm that the present dataset is suitable for EFA. The KMO test (KMO test score = 0.646) and the Bartlett’s Test of Sphericity (χ2 = 23404.929, df = 91, p < 0.001) indicated the factorability and sampling adequacy of the current dataset. Step-2 is generating the factor extraction. Maximum likelihood, which is considered the most common extraction method, was selected for this step. Step-3 is applying factor rotation. The oblique rotation, which allows factors to be correlated, was applied to explore if correlations existed among the extracted factors. With the initial results, moderate positive correlations were found among the extracted factors, so promax was selected as the rotation method. By the end of this step, we generate an initial solution, and all factors (representing the latent dimensions of grammatical complexity) were extracted if their eigenvalues are greater than 1. Step-4 is setting the factor loadings. As factor loadings above 0.30 or 0.40 are considered “rule of thumb,” we suppressed all small coefficients with absolute value less than 0.40 based on a recent suggestion (Furr, 2018). Step-5 is comparing different solutions to achieve the most suitable EFA model. We selected a best mode based on three criteria: (1) the percentage of the total variance explained, (2) the number of items loading on each factor and the values of factor loading coefficients, and (3) fewer or no cross-loadings or error loadings.

## Results

### Research Question-1

Based on the EFA, a four-factor model was found to have the best fit. The four-factor model is based on the 14 grammatical complexity measures, which explains 86.506% of the total variance of the dataset. To be specific, the four factors are Factor 1 (27.853%), Factor 2 (35.816%), Factor 3 (14.112%), and Factor 4 (8.725%). Table 2 shows the pattern matrix of the four-factor model and our interpretation of the four factors. In terms of the matrix pattern:

1. Six complexity measures (DC/C, DC/T, CT/T, C/T, VP/T, C/S) show significant positive loadings on Factor 1.
2. Four measures (CP/T, CP/C, CN/C, CN/T) show significant loadings on Factor 2.
3. Three complexity measures (MLT, MLC, MLS) have significant loadings on Factor 3.
4. Three complexity measures (T/S, C/S, MLS) have significant loadings on Factor 4.

**TABLE 2:** Four-factor solution: Pattern matrix.

Dimensions	Measure	Description	Factor 1	Factor 2	Factor 3	Factor 4
Clausal subordination	DC/C	Dependent clauses per clause	1.073			
	DC/T	Dependent clauses per T-unit	1.055			
	CT/T	Complex T-units per T-unit	0.948			
	C/T	Clauses per T-unit	0.945			
	VP/T*	Verb phrases per T-unit	0.787			
Phrasal construction	CP/T*	Coordinate phrases per T-unit		1.023		
	CP/C*	Coordinate phrases per clause		1.011		
	CN/C*	Complex nominals per clause		0.505		
	CN/T*	Complex nominals per T-unit		0.457		
Global length unit	MLT	Mean length of T-unit			0.967	
	MLC	Mean length of clause			0.902	
	MLS	Mean length of sentence			0.681	0.438
Others	T/S*	T-units per sentence				1.012
	C/S	Clauses per sentence	0.559			0.619

“*” Marks the complexity measures that are discussed in detail.

Two noteworthy points should be mentioned. First, there are two cross-loading measures in the pattern matrix. MLS has cross loading on Factor 3 (0.681) and Factor 4 (0.438), and C/S has cross loading on Factor 1 (0.559) and Factor 4 (0.619). Acknowledging MLS and C/S may measure different grammatical dimensions, they are categorized to factors with higher loadings: Factor 3 for MLS and Factor 4 for C/S. Second, a well-defined factor should ideally have at least three significantly loaded variables, but two highly correlated variables also have the potential to reasonably define a reliable factor (Tabachnick and Fidell, 2012). Therefore, we decided to keep Factor 4 (others) in the EFA model.

### Research Question-2

The EFA model was compared with the model in Lu (2011). Table 3 demonstrates the EFA model and Lu’s (2011) model are mostly similar; however, relevant measures are marked with an “*” to show notable differences. There are three differences which are all noteworthy: (1) the measures of coordinate phrases (i.e., CP/T, CP/C) are categorized into “coordination” in Lu (2011), whereas the EFA model shows that they are categorized into “phrasal construction” accompanied by the other two measures, CN/C and CN/T. (2) VP/T is categorized as “clausal subordination” in the EFA model but as a particular structure in Lu (2011). (3) T/S and C/S are categorized as “others.” T/S — labeled as “coordination” in Lu (2011) — does not load with the other two coordinate measures (CP/C, CP/T). Please see Table 3 for details.

**TABLE 3:** Comparison of the EFA model and the model in Lu (2011).

Measures	Dimensions in the EFA model	Grammatical type in Lu (2011)
DC/C	Clausal subordination	Subordination
DC/T		Subordination
CT/T		Subordination
C/T		Subordination
VP/T*		Particular structures
CP/T*	Phrasal construction	Coordination
CP/C*		Coordination
CN/C*		Particular structures
CN/T*		Particular structures
MLT	Global length unit	Length of production
MLC		Length of production
MLS		Length of production
T/S*	Others	Coordination
C/S		Sentence complexity

“*” Marks the complexity measures that are discussed in detail.

## Discussion

### Research Question-1

Based on loading patterns of the 14 measures in the EFA model, four dimensions are proposed: “clausal subordination,” “phrasal construction,” “global length unit,” and “others.” The four dimensions align with the claim that grammatical complexity is a multidimensional construct (Norris and Ortega, 2009). First, the measures in “clausal subordination” are primarily clausal features over T-units (e.g., DC/T). As T-units refers to the main clause and all the dependent clauses attaching to this main clause, the T-unit-based measures are subordinate-driven (Bulté and Housen, 2012). Second, “phrasal construction” includes phrasal structures (i.e., coordinate phrases and complex nominals). These features are reasonably loaded on the dimension of phrasal construction because they are mostly associated with phrasal features. From a grammatical perspective, the phrasal construction includes two different types of phrases – coordinate phrase construction and nominal phrase construction. Coordinate phrases can include coordinate adjectival, verbal and/or adverbial phrases, and nominal phrases can include noun-headed phrases, pronoun-headed phrases, and others.

Third, “global length unit” contains three length-based measures. Length tends to be the most global measurement unit because nearly all grammatical features can extend the length of a sentence, both phrases and clauses (Lan et al., 2019). We were not surprised to find the three length-based measures (i.e., MLT, MLC, and MLS) load on the same dimension. Fourth, “others” is a debatable dimension in our EFA model, for theoretically T/S and C/S do not measure exactly the same type of grammatical structure. T/S is designed to measure coordinate clauses, whereas C/S is designed to analyze sentence complexity in general (Ortega, 2003).

A tentative interpretation of “others” is that coordinate clauses (T/S) largely contribute to sentence complexity (C/S) in our corpus, the L2 writing from the BAWE corpus. In terms of the design of the two measures, both T/S and C/S have the same denominator (i.e., sentence), and they are only different in numerators (i.e., T-unit and clause). The results show that in our specific corpus, the number of T-units within a sentence is similar to the number of clauses within a sentence. Based on previous studies regarding grammatical complexity in L2 writing, phrasal elaboration is the major grammatical characteristics of academic writing rather than clausal subordination (e.g., Biber et al., 2011). A T-unit may contain one clause rather than two clauses in our corpus of L2 academic writing. For other corpora, these two measures may not be likely to be grouped in the same factor. Thus, we interpret this dimension as “others,” and a further investigation can be conducted to validate this tentative interpretation.

### Research Question-2

In terms of the three differences, the interpretations are provided in this section accordingly. First, it is reasonable to classify the four measures into “phrasal construction” because the two measures of complex nominals (CN/C, CN/T) are primarily based on complex noun phrases (i.e., nouns with pre- and post-modifiers). In the EFA model, the loadings of the four measures tend to be bifold, although they are all significant: CP/T (1.023) and CP/C (1.011) with noticeably higher loadings than CN/C (0.505) and CN/T (0.457). This shows both a coordinate-phrase sense of phrasal construction and a noun-phrase sense of phrasal construction. Our data is primarily based on the L2 academic writing, we consider the coordinate-phrase sense of phrasal construction is largely based on coordinate noun phrases. This is due to the empirical evidence in previous large-scale corpus studies that noun phrases are highly frequent in academic writing (Lan and Sun, 2019).

Second, while admitting that VP/T is a specific measure based on verb phrases, we also think that this measure could be related to subordinate clauses. Verb phrases (VPs) are defined as verb nodes that are immediately dominated by clauses in the L2SCA. It can be considered that the number of verb phrases tends to be correlated with the number of clauses, because it is required for a clause to include a main verb in English. Thus, VP/T could indicate clausal subordination as well. Similar studies could be conducted to examine whether our assumption is borne out with other corpora.

Third, T/S primarily measures coordinate clauses, whereas CP/T and CP/C measure coordinate phrases. This difference is captured by the EFA. As a result, the EFA model also indicates the importance of measuring coordinate clauses and phrases as two separate types of grammatical structures. CP/C and CP/T perform differently from T/S when they are used to measure grammatical complexity in L2 writing. In addition, C/S is the trickiest measure in the EFA model, which has close loadings on “clausal subordination” (0.559) and “others” (0.619). We ultimately decided to put this measure in “others” because (1) it has a higher loading in “others” and (2) it is an omnibus measure of sentence complexity in general instead of subordinate clauses.

## Conclusion and Limitation

Following the existing research trend of investigating the multidimensionality of linguistic complexity, we conducted an EFA to explore the latent dimensions of grammatical complexity in L2 writing in particular. Our EFA model includes four factors, being interpreted as global length unit, clausal subordination, phrasal construction, and others. Some differences can be found between the EFA model and Lu’s (2011) categorization of the measures. However, these differences are not unreasonable. For instance, two coordinate phrases measures (CN/C, CN/T) were loaded on phrasal construction in our EFA model instead of being categorized as coordination measures as in Lu (2011). We call for more EFA studies on how latent components of grammatical complexity are represented with existing measures.

At the end, we need to acknowledge several limitations of this study. First, in order to get enough samples for the EFA, we did not control some factors that can influence grammatical complexity in L2 writing, such as L1 backgrounds and written genres. A further EFA can be conducted to triangulate our findings with the application of Lu (2011)’s 14 measures in more controlled research contexts. Second, it is also suggested to have a qualitative linguistic analysis on the L2 BAWE files to see how to interpret the fourth dimension, “others.” This dimension includes two different types of measures focusing on coordinate clauses and sentence complexity in general. So far it is still unclear how to interpret this dimension. Third, it is important to mention that L2 writing is a developmental process. The latent dimensions of grammatical complexity in our EFA modal (e.g., clausal subordination, phrasal construction) would play different roles in analyzing student writing at different institutional levels. With the L2 data in the BAWE corpus, we attempted to address this issue by running four different EFAs for four different institutional levels (e.g., first/second/third year of undergraduate levels, and graduate level). However, the insufficient samples prevented us from addressing our research questions from a developmental perspective. Fourth, as the data is limited in the L2 BAWE corpus, we didn’t perform CFA to validate our EFA model. We plan to complete the process by adding CFA to our future research when a larger L2 corpus is available. Last, it is important to mention that mean length of clause (MLC) has been interpreted as a phrasal-level measure in Norris and Ortega (2009). The length of clauses is primarily influenced by the phrases within the clauses. However, based on the EFA results, the MLC taps into “global length unit,” consistent with Lu’s (2011) categorization. We need to admit that the same measures may play different roles in measuring grammatical complexity. This is also worth of being further discussion in the future.

## Data Availability Statement

The datasets presented in this article are not readily available because they are subject to ongoing research. Requests to access the datasets should be directed to GL (gelan4@cityu.edu.hk).

## Author Contributions

GL worked on the EFA model interpretation and theoretical discussion. XL worked on the EFA analysis. QZ worked on corpus and dataset building. All authors contributed to the article and approved the submitted version.

## Conflict of Interest

The authors declare that the research was conducted in the absence of any commercial or financial relationships that could be construed as a potential conflict of interest.

## Publisher’s Note

All claims expressed in this article are solely those of the authors and do not necessarily represent those of their affiliated organizations, or those of the publisher, the editors and the reviewers. Any product that may be evaluated in this article, or claim that may be made by its manufacturer, is not guaranteed or endorsed by the publisher.
